# Reducing blood culture contamination in the ED: impact of Kurin Lock® implementation

**DOI:** 10.1016/j.infpip.2026.100541

**Published:** 2026-04-13

**Authors:** E. Browne, M. Russell, G.K. Muniyappa, M. Abbas, P. Ratenavelu, F. Fitzpatrick, O. Keane, H. McDermott

**Affiliations:** a Department of Clinical Microbiology, Beaumont Hospital, Dublin, Ireland; b Department of Clinical Microbiology, Royal College of Surgeons in Ireland, Dublin, Ireland; c Department of Infection Prevention and Control, Beaumont Hospital, Dublin, Ireland; d Department of Emergency Medicine, Beaumont Hospital, Dublin, Ireland

**Keywords:** Blood culture contamination, Emergency department, Kurin lock®, Initial specimen diversion device, Antimicrobial stewardship

## Abstract

**Background:**

Blood culture contamination (BCC) poses significant clinical and financial challenges in hospital settings. Guidelines recommend BCC rates remain below 3%; however, in the first quarter (Q1) of 2024, our tertiary hospital emergency department (ED) reported a median rate of 10%.

**Aim:**

The aim of the present study was to evaluate the impact of the Kurin Lock® device on reducing BCC rates in the ED. The device diverts the initial 0.15 mL of blood, potentially containing skin flora contaminants.

**Methods:**

An eight-week pilot of the Kurin Lock® device was conducted from May to July 2024. ED clinical staff received training prior to implementation. Blood cultures collected using Kurin Lock® were compared with those obtained via the standard method. Contamination was defined by the clinical microbiology team as isolation of organisms considered clinically insignificant and likely derived from skin flora. Data were extracted from the hospital’s surveillance system.

**Findings:**

A total of 768 blood cultures were collected. Contamination occurred in 2.7% (*N* = 6/221) of blood cultures obtained using Kurin Lock® compared with 10.4% (*N* = 57/547) of blood cultures obtained using the standard method (*P* = 0.0004), representing a 74% relative reduction. Staff adherence to device use averaged 28.5%.

**Conclusion:**

Use of the Kurin Lock® was associated with a significant reduction in BCC, achieving rates below the 3% recommended standard. These findings highlight the potential benefits of improving patient outcomes and healthcare costs. Ongoing success will depend on increasing staff engagement and integration into routine practice.

## Introduction

Blood culture is the primary diagnostic tool for detecting bloodstream infection [1]. Blood culture contamination (BCC), typically from the introduction of skin flora during sample collection, can yield false positive results [1]. BCC is particularly common in emergency departments (EDs), where rapid patient turnover and urgent care increase risk [1]. BCC complicates interpretation, often leading to unnecessary investigations**,** inappropriate antimicrobial therapy, prolonged hospital length of stay (LOS), and increased microbiology laboratory workload, all driving healthcare costs [[2], [3], [4]]. A Northern Ireland study estimated an additional cost of £5000 per contaminated blood culture [2].

BCC-related costs include staff time, consumables, antimicrobials, and hospital resources. A previous unpublished time-in-motion analysis conducted in our laboratory indicated that processing a contaminated blood culture requires approximately 56 min of laboratory time per bottle over two days, excluding molecular and susceptibility testing. Clinicians also spend time on reassessment, repeat sampling, and antimicrobial administration. Consumable costs encompass culture media, microscopy slides, and biochemical testing materials and reagents, with additional expenses incurred when rapid molecular testing is requested. In an unpublished local 12-week audit conducted at our institution, 14 (12.2%) of 115 contaminated blood cultures underwent molecular testing.

Antimicrobial misuse is common: up to 41% of patients with BCC receive unnecessary antimicrobials, with vancomycin prescribed in up to 84% [3,5]. In contrast, our unpublished local 12-week audit showed vancomycin use in only eight (7%) of 115 contaminated blood cultures, likely reflecting clinical microbiology team (CMT) input and molecular diagnostics. Even short inappropriate courses can cause adverse events, catheter infections, and *Clostridioides difficile* infection, leading to additional complications [4]. BCC also prolongs LOS. Northern Ireland data reported a median LOS of 13 days for patients with BCC compared with eight days for true negative blood cultures [2]. Similarly, US data showed an LOS of seven days for BCC vs five days for true negatives [3]. In our unpublished local audit, patients with BCC had a mean LOS of 10.5 days, compared with a hospital mean of 8.7 days for the same period, without adjustment for confounders. Because of these clinical and financial consequences, international guidance recommends BCC rates below 3%, ideally 1% with best practice [4,6]. Many hospitals, particularly EDs, exceed these thresholds [1]. In the first quarter (Q1) of 2024, Beaumont Hospital’s ED median BCC rate was 10%.

**Figure 1:**
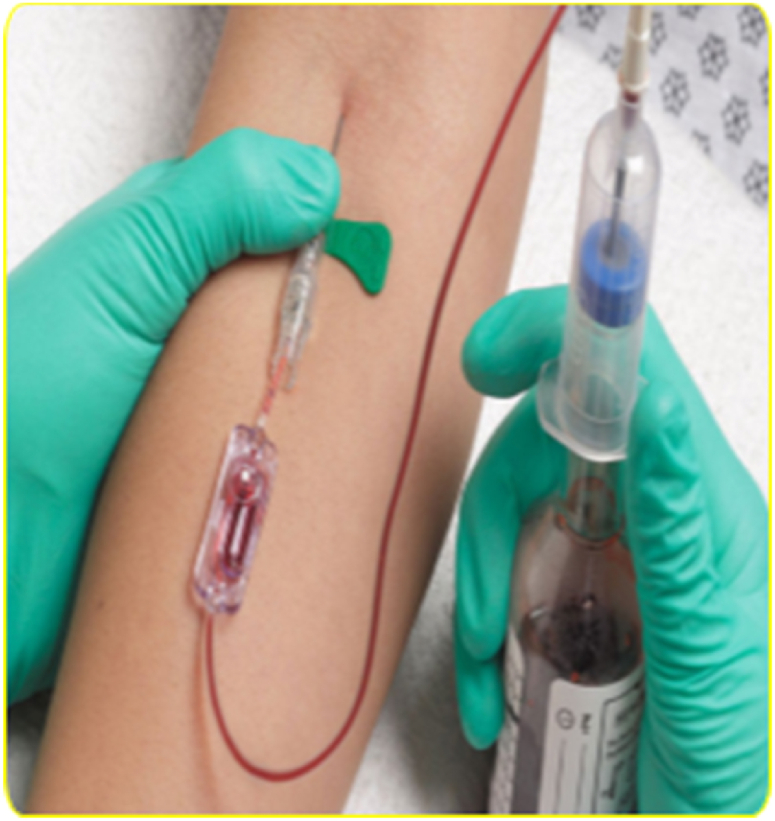
Kurin Lock® device with integrated diversion chamber to reduce skin contamination during blood culture collection. Image reproduced with the permission of Iskus Health.

Initial specimen diversion devices (ISDDs) such as the Kurin Lock® (Becton Dickinson, USA) offer a potential solution. The device diverts the initial 0.15 mL of blood, most likely to contain contaminants, into a U-shaped flash chamber (Figure 1). The remaining sample is collected into standard blood culture bottles. The device is easy to use, requires minimal training, and costs approximately £20 [7]. Evidence from US and Irish studies supports the effectiveness of ISDDs in reducing BCC rates [[7], [8], [9]]. At an Irish tertiary hospital, contamination dropped from 18.4% to 4.9% with Kurin Lock® and rebounded after withdrawal [9].

Given our high baseline BCC rate, we conducted an eight-week pilot to evaluate the impact of Kurin Lock® on BCC in our ED.

## Methods

### Setting

An eight-week pilot study was conducted in Beaumont Hospital’s ED, Dublin, Ireland, from 8^th^ May to 2^nd^ July 2024. Beaumont Hospital is an 851-bed tertiary teaching hospital and national referral centre for neurosurgery and renal transplantation. Blood cultures are processed on-site in a continuously operating clinical laboratory. In 2024, the ED recorded 66,608 attendances.

### Intervention

The Kurin Lock® device (venipuncture and peripheral venous catheter [PVC] connector device) was introduced for blood culture collection for the duration of the pilot.

Prior to implementation, 130 ED staff (36 doctors, 94 nurses) attended a 15-min training session delivered by an Iskus Health representative.

### Study design

This was a single-centre, prospective, non-randomised implementation pilot study. The Kurin Lock® device was introduced into routine clinical practice, and its use was voluntary following structured training provided to ED staff. No patient-level selection was applied. Allocation to either the Kurin Lock® or standard blood culture collection was determined solely by the device chosen by staff at the time of blood culture collection. All ED patients for whom blood cultures were indicated (as determined by the ED clinical team) during the study period were eligible for inclusion. The primary outcome measure was BCC rate, compared between the two collection methods.

### Standard-of-care venipuncture

Routine blood cultures were obtained via peripheral venipuncture using an aseptic non-touch technique, in accordance with the hospital's standard operating procedure (SOP) for peripheral blood culture collection. Following removal of the protective caps, the membrane of each blood culture bottle was disinfected using a swab containing 2% chlorhexidine gluconate in 70% isopropyl alcohol and allowed to air-dry. Skin antisepsis was then performed with a separate 2% chlorhexidine gluconate in 70% isopropyl alcohol swab, applied using a friction scrub technique for 30 s. The venipuncture site was allowed to dry completely before proceeding. This skin preparation and aseptic protocol was applied to both standard collection and Kurin Lock® procedures.

Standard-of-care venipuncture was performed using a butterfly needle attached to a BD Vacutainer® (Becton Dickinson, USA) blood collection set. A volume of 8–10 mL of blood was inoculated into each aerobic and anaerobic blood culture bottle, which was then transported to the microbiology laboratory for incubation.

### Data collection

For each blood culture obtained using the Kurin Lock®, ED staff completed a form with date, signature, and patient label (printed at registration) for tracking. Completed forms were deposited at four ED collection points and retrieved twice weekly by designated ED doctors and CMT members. Patient identifiers were anonymised and recorded in a Microsoft Excel database with blood culture collection date/time and collector’s name. Incomplete forms were excluded.

### Microbiology workup

Blood cultures were collected using standard aerobic and anaerobic BD BACTEC™ bottles (one set comprising one aerobic and one anaerobic bottle) and incubated in the BD BACTEC™ FX automated continuous-monitoring blood culture system (Becton Dickinson, USA) in accordance with the manufacturer’s instructions. Bottles signalling positive underwent Gram staining and were subsequently subcultured on to appropriate solid media. Organisms were identified using matrix-assisted laser desorption ionisation–time-of-flight mass spectrometry (MALDI-TOF MS) with the MALDI Biotyper® sirius system (Bruker, Germany).

### Outcomes and statistical analysis

Blood culture volumes and contamination data were extracted from the hospital’s surveillance database, which receives automated weekly laboratory feeds. All positive blood cultures were reviewed by the CMT, to determine clinical significance and source. ED-specific extracts were exported to Excel. Contamination was defined by the CMT as the recovery of organisms typically associated with skin flora (e.g. coagulase-negative staphylococci, *Micrococcus* species, non- anthracis *Bacillus* species, *Corynebacterium* species, *Cutibacterium acnes*, and viridans group streptococci), in the absence of clinical evidence supporting true bacteraemia. Classification incorporated a number of factors including the organism identified, the number of blood culture bottles and sets that were positive, time to positivity, the presence of prosthetic material, and the overall clinical context. The BCC rate was calculated as the number of blood culture sets classified as contaminants divided by the total number of blood culture sets collected during the study period. Rates were determined separately for blood cultures collected using the Kurin Lock® and those collected using the standard method.

Contamination rates were compared between Kurin Lock® and standard collection using Pearson's *χ*^2^ test in Excel, with exact *P*-values calculated via the CHIDIST function. Relative risk (RR) and corresponding 95% confidence intervals (CIs) were calculated from the 2 × 2 contingency table. Statistical analyses were performed in Microsoft Excel using a two-sided significance level of α = 0.05.

## Results

During the eight-week pilot, 768 blood cultures were collected in Beaumont Hospital’s ED, 28.8% (*N* = 221) using the Kurin Lock® and 71.2% (*N* = 547) by the standard method. Contamination occurred in 2.7% (*N* = 6/221) of cultures obtained using the Kurin Lock® compared with 10.4% (*N* = 57/547) using the standard method, representing a 74% relative reduction in contamination risk (RR 0.26, 95% CI 0.11–0.60; *P* = 0.0004).

**Figure 2:**
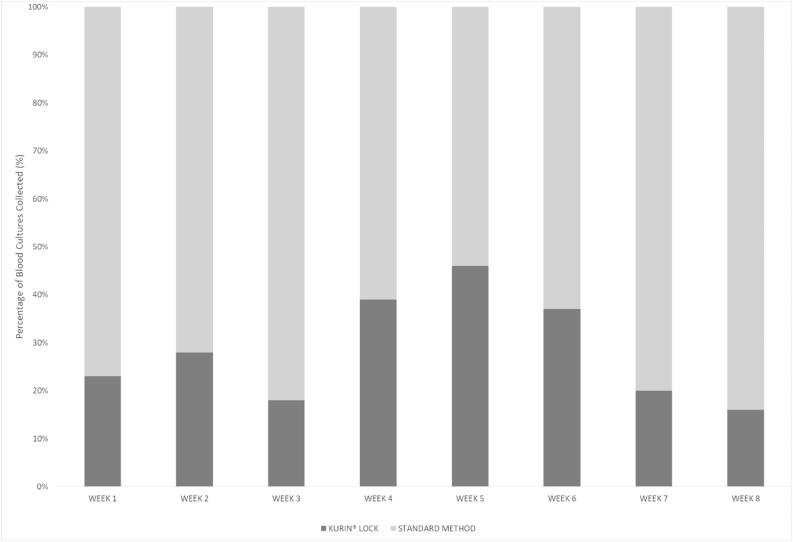
Weekly proportion of emergency department (ED) blood cultures collected using Kurin Lock® vs standard method. Kurin® Lock mean adherence 28.5% (range: 16.5%–46.2%).

Staff adherence to Kurin Lock® use averaged 28.5% across the pilot period (range: 16.5%–46.2%) (Figure 2). Of 130 trained staff, 74.6% (*N* = 97) used the device at least once during the trial period, 83.5% (*N* = 81) used it fewer than three times, while only 2.1% (*N* = 2) used the device ten or more times.

## Discussion

Implementation of the Kurin Lock® in Beaumont Hospital’s ED was associated with a 74% reduction in BCC, achieving a contamination rate of 2.7% for cultures collected using the device – below the internationally recommended 3% threshold [6]. These findings further strengthen the expanding evidence base supporting the use of ISDDs, such as the Kurin Lock®, to improve blood culture quality. They also provide contemporary, ED-specific, real-world data demonstrating the device’s effectiveness within an Irish context characterised by a high baseline contamination rate and considerable operational pressures. Comparable reductions have been reported at Hartford Hospital, USA (74.1% reduction from a baseline of 1.71%), and St Vincent’s University Hospital, Ireland (73.4% reduction from a baseline of 18.4%) [8,9].

Although downstream effects were not measured, reduced contamination is expected to lower laboratory and clinical workloads, consumable use, and unnecessary antibiotic prescribing, thereby reducing costs and adverse events. Economic modelling indicates cost savings when ISDDs are used in EDs with contamination rates above 9% [7]. Reflecting this, the National Institute for Health and Care Excellence now recommends its adoption in these settings [7].

Despite these benefits, staff adherence was low (28.5%). Informal, non-systematically collected staff feedback suggested several barriers: inconsistent availability of device types (with a preference for PVC connector devices), time constraints during busy shifts, and practical constraints linked to the ED's older infrastructure. Verbal encouragement to use the Kurin Lock® device was provided throughout the pilot period. Adherence during the first three weeks was low, prompting increased efforts to promote device use during weeks 4 and 5. This included reinforcement on the clinical floor, daily reminders at clinical handover and via the team WhatsApp group, as well as reminders incorporated into routine ED teaching sessions. These combined interventions likely contributed to the observed increase in device uptake. No additional formal re-training was undertaken.

The decline in device use during weeks 6–8 is more challenging to explain definitively. Staffing patterns may have played a contributory role. Beaumont Hospital’s ED relies partly on temporary agency (locum) staff, who had not received training in device use, reflecting the inherent dependence of a 24-h service on rotating staff. Some blood cultures labelled as ED samples may have been collected by non-ED staff who were similarly untrained, representing a potentially important but unquantifiable source of reduced adherence. The pilot was also concluded shortly before the annual non-consultant hospital doctor changeover in Ireland, which may have influenced engagement with the intervention, although this cannot be confirmed. Adherence may additionally have been underestimated due to the manual data collection, which captured only Kurin Lock® blood cultures accompanied by a completed form. Finally, an unseasonably high number of ED presentations during the later part of the pilot may have further influenced device uptake.

Adherence challenges have been reported with other ISDDs, such as Steripath®, suggesting that uptake barriers are not unique to Kurin Lock® [10]. Engaging the hospital’s intravenous cannulation (IVC) team may improve uptake; however, staffing limitations must be considered.

Limitations of this pilot include its single-centre, short-duration design, which limits generalisability and does not account for seasonal variation. Contamination definitions relied on clinical judgement that may differ across institutions, and low adherence with limited device use introduces potential selection bias. Contamination outcomes were not recorded by device type, limiting interpretation of device-specific effects. Despite these limitations, this study was conducted in a busy high-volume ED during a period of high staff turnover, reliance on temporary staff, and substantial workflow pressures, offering practical implementation insights that complement findings from controlled settings. Blood cultures were collected across multiple shifts and staff groups following structured training. Contamination outcomes were obtained from routinely updated surveillance databases, allowing objective comparison with standard practice. Although the number of contaminated samples was small (*N* = 63), potentially constraining causal inference, the association between collection method and contamination remained statistically significant, suggesting real-world evidence of intervention impact consistent with other published evaluations of Kurin Lock®.

In conclusion, Kurin Lock® implementation in our ED was associated with a significant reduction in BCC, achieving rates below international targets. While downstream outcomes were not measured, the magnitude of BCC reduction suggests meaningful clinical and operational benefits. Following completion of the pilot, permanent adoption of the device was approved, and implementation commenced in March 2025. The intervention has since been embedded into routine clinical practice, and ongoing quality improvement initiatives are in place to support sustained adherence and optimise implementation.

Sustained impact will require consistent device use, supported by education, training, audit, and engagement of key stakeholders, including hospital management, emergency medicine clinicians, IVC team, procurement, and device representatives. Reliable supply chains and workflow integration are essential. Expansion to other areas, such as intensive care and inpatient wards, should be considered, supported by hospital-wide training to ensure consistent device use. Further studies are warranted to evaluate effectiveness in these settings.

## CRediT authorship contribution statement

**E. Browne:** Writing – review & editing, Writing – original draft, Investigation, Data curation. **M. Russell:** Writing – review & editing, Writing – original draft, Visualization, Formal analysis, Data curation. **G.K. Muniyappa:** Writing – review & editing, Visualization, Investigation. **M. Abbas:** Writing – review & editing, Investigation. **P. Ratenavelu:** Writing – review & editing, Investigation. **F. Fitzpatrick:** Writing – review & editing, Supervision, Investigation. **O. Keane:** Writing – review & editing, Supervision, Resources, Project administration, Methodology, Investigation, Conceptualization. **H. McDermott:** Writing – review & editing, Supervision, Resources, Project administration, Methodology, Investigation, Conceptualization.

## Ethical approval

Ethical approval was not required for this study.

## Funding sources

The authors received no external funding for this study.

## Conflict of interest statement

Kurin Lock® devices were procured from Iskus Health (Dublin, Ireland), and the hospital received an additional product unit from the company at no extra cost. Iskus Health had no role in the study design, data collection, analysis, interpretation, or manuscript preparation.
